# Identification and validation of plasma protein biomarkers as therapeutic targets in acute myeloid leukemia: an integrative multi-omics study

**DOI:** 10.3389/fimmu.2025.1659811

**Published:** 2025-10-22

**Authors:** Linhui Hu, Qingqing Luo, Ya Liao, Zhimin Zhai, Yangyang Ding, Yan Fei

**Affiliations:** ^1^ Department of Hematology, The Second Affiliated Hospital of Nanchang University, Nanchang, China; ^2^ Jiangxi Provincial Key Laboratory of Hematological Diseases (2024SSY06052), Nanchang, Jiangxi, China; ^3^ Department of Hematology, The Second Affiliated Hospital of Anhui Medical University, Heifei, China

**Keywords:** acute myeloid leukemia, Mendelian randomization, plasma proteins, therapeutic targets, multi-omics

## Abstract

**Introduction:**

Acute myeloid leukemia (AML) remains a therapeutic challenge due to its high relapse rate and limited treatment options. This study aimed to identify and validate novel circulating protein biomarkers with causal roles in AML pathogenesis using an integrative multi-omics approach.

**Methods:**

We performed proteome-wide Mendelian randomization (MR) analyses using protein quantitative trait locus (pQTL) data from two large-scale proteomic studies (deCODE and UK Biobank Pharma Proteomics Project) and genome-wide association study (GWAS) data from two cohorts (FinnGen and UK Biobank). Single-cell RNA sequencing was used to analyze the expression patterns of candidate proteins in hematopoietic progenitor and immune cells. Plasma protein levels were experimentally validated via ELISA in AML patients and healthy controls, and their dynamic changes relative to disease status were assessed. Drug repurposing analysis and phenome-wide association studies (PheWAS) were conducted to evaluate potential therapeutic agents and their safety profiles.

**Results:**

Three independent MR analyses identified TNFAIP8, TCL1A, and WFDC1 as risk factors for AML, while TNFSF8 was identified as a protective factor. Single-cell RNA sequencing revealed distinct expression patterns of these proteins within hematopoietic progenitor and immune cells, suggesting roles in microenvironmental dysregulation. ELISA validation confirmed elevated plasma levels of TNFAIP8, TCL1A, and WFDC1 and reduced levels of TNFSF8 in AML patients compared to healthy controls. Dynamic changes were observed for TNFAIP8 and TNFSF8, supporting their potential for disease monitoring. Drug repurposing analysis prioritized 13 candidates targeting these proteins, including FDA-approved agents, and PheWAS supported their safety.

**Conclusion:**

This study provides the first genetic evidence supporting the causal roles of TNFAIP8, TCL1A, WFDC1, and TNFSF8 in AML, offering new insights for targeted therapy development and biomarker-based disease monitoring.

## 1 Introduction

Acute myeloid leukemia (AML), the most common adult leukemia, is a heterogeneous hematological malignancy characterized by clonal myeloid cell proliferation (1). With an incidence rate of 4.3 per 100,000 individuals and a median diagnosis age of 65 years (2), AML poses a significant threat to public health, causing over 80,000 annual deaths globally, a figure expected to double within the next 20 years (3). Despite advances in targeted therapies, the relapse rate remains as high as 60-80% (4), highlighting the urgent need to elucidate AML pathogenesis and identify novel therapeutic targets.

Proteomic advances have enabled the identification of over 5,000 plasma proteins, which are essential components of circulating blood and play critical roles in various physiological and pathological processes. Some of these proteins serve as sensitive biomarkers for inflammation, infection, and systemic diseases, offering tools for early diagnosis, monitoring, and potential therapeutic targets (5, 6). To uncover potential links between specific proteins and leukemogenesis, several cross-sectional studies have investigated the differences in plasma protein levels between AML patients and healthy controls. Bai et al. (7)reported elevated levels of UBA1, FGA, and PF4 proteins in AML patients, which decreased after complete remission. Zheng et al. (8) identified 14 plasma proteins with abnormal expression in AML patients and found that serum lectin was notably associated with the efficacy of standard chemotherapy. Zhang et al. (9) demonstrated that ICAM2 serves as a strong prognostic marker for survival in intermediate-risk AML patients, regardless of whether they undergo bone marrow transplantation. However, the reliability of these observational studies is limited by their susceptibility to bias and reverse causation.

Mendelian randomization (MR) uses genetic variants as instrumental variables to infer causal effects, reducing susceptibility to confounding and reverse causation biases. Previous MR studies have demonstrated causal links between gut microbiota, immune cell phenotypes, vitamin D, branched-chain amino acids, and AML development (10–13). Proteome-wide MR studies investigate genetically determined circulating protein concentrations and their potential roles in disease etiology, having been successfully applied to colorectal, breast, and lung cancers (14–16). Regretfully, to date, no study has utilized MR to investigate the relationship between plasma proteins and AML.

To address this gap, we performed a systematic proteome-wide MR analysis aimed at identifying novel therapeutic targets for AML and providing new insights for the development of future AML treatments. By leveraging large-scale genetic and proteomic data, our study sought to uncover causal associations between circulating proteins and AML risk, informing the development of targeted therapies and risk prediction models. This integration of proteomics and causal inference methods represents a promising approach for advancing our understanding of AML pathogenesis and identifying actionable targets for intervention.

## 2 Methods

### 2.1 Study design

**Figure 1:**
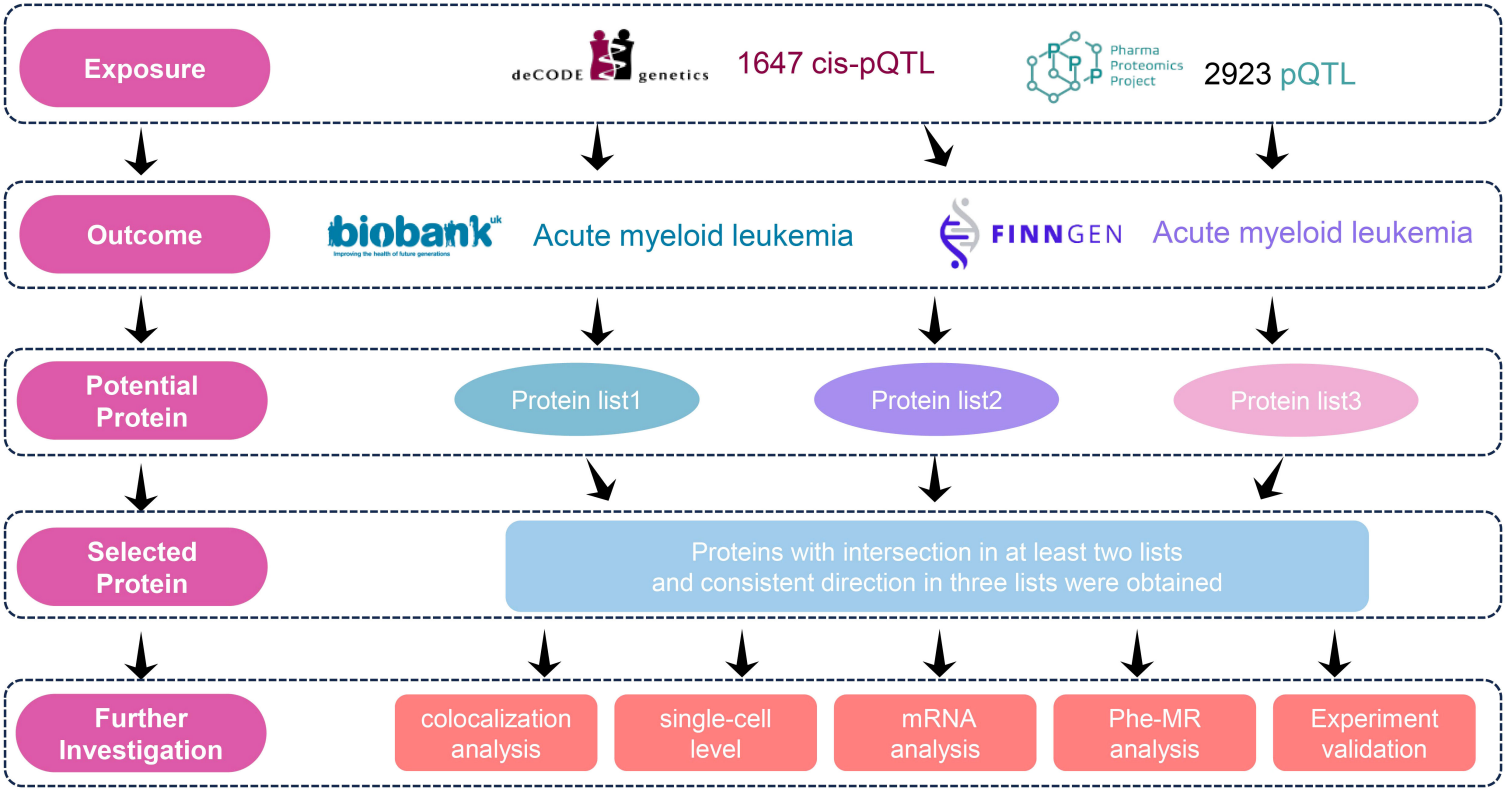
Flowchart of the study design.

In this study, we selected protein quantitative trait loci (pQTL) data from two large-scale plasma proteomics studies (deCODE Genetics study and the UK Biobank Pharma Proteomics Project (UKB-PPP)) as exposure variables and used genome-wide association study (GWAS) data from two cohorts (UK Biobank and the FinnGen cohort) as outcome measures to investigate the potential causal relationship between plasma proteins and AML risk. To avoid bias caused by population overlap (UK biobank and UKB-PPP), we conducted three independent MR analyses (deCODE to FinnGen, deCODE to UK Biobank, and UKB-PPP to FinnGen). We identified proteins that were nominally significant in at least two analyses and aligned in direction in three analyses as potential causal biomarkers. To assess the robustness of our findings, Bayesian colocalization analysis was performed. To further examine the expression patterns of the candidate biomarker genes across different cell types and reveal their cell-specific roles in AML, we utilized single-cell RNA sequencing (scRNA). Meanwhile, we analyzed the expression patterns of the candidate biomarker genes in AML subtypes defined by ELN and WHO guidelines (17, 18). The candidate proteins were then experimentally validated using enzyme-linked immunosorbent assay (ELISA). Additionally, a phenome-wide association study (PheWAS) was conducted to confirm the safety of the identified targets. Finally, we carried out druggability assessments to evaluate the potential of the identified plasma proteins as therapeutic targets. The detailed study design workflow is illustrated in **Figure 1**.

### 2.2 Data sources and selection of IVs

GWAS data for AML were obtained from two datasets: the UK Biobank (cases = 220, controls = 456,128) and the FinnGen cohort (cases = 244, controls = 314,192). pQTL data were sourced from two studies: the Icelandic deCODE Genetics study (deCODE) and the UK Biobank Pharma Proteomics Project (UKB-PPP). The deCODE study provided pQTL data for 4,907 plasma proteins, measured in 35,559 Icelandic individuals using the SomaScan v4 platform. The UKB-PPP dataset included detailed pQTL mapping for 4,572 proteins, measured in 54,219 participants from the UK Biobank using the Olink platform.

To identify pQTLs, we applied the following criteria: To identify pQTLs, we applied the following criteria (19): (1) The SNP was located within a vicinity of ±1 Mb around the gene region (cis-acting pQTLs); (2) The SNP-protein association reached a genome-wide significant threshold of P < 5 × 10^-8^; (3) The independence assumption was satisfied through linkage disequilibrium clumping (r² < 0.001); (4) Palindromic SNPs were excluded to avoid allele orientation ambiguity; (5) The SNPs with an F-statistic < 10 were excluded to ensure strong instrument strength and minimize weak instrument bias. Detailed results for each protein, including the number of instruments, variance explained (R²), and F-statistic are provided in the **Supplementary Table 1**.

### 2.3 MR analysis

In this study, we conducted MR analyses using plasma proteins as exposure variables and AML as the outcome variable. The selection criteria for pQTLs strictly adhered to the standards described earlier. For proteins with three or more SNPs, the inverse-variance weighted (IVW) method was used as the primary approach. In addition, sensitivity analyses, including MR-Egger, weighted median, and weighted mode were conducted to evaluate pleiotropy and heterogeneity. For proteins instrumented by a single SNP, the Wald ratio method was applied. And for proteins instrumented by two or less SNP, sensitivity analyses were not performed (20). We further applied Steiger filtering to verify the direction of causality (exposure to outcome). Furthermore, to rule out potential reverse causation, we performed reverse MR analysis for all significant proteins identified in the forward analysis, using AML as the exposure and the respective protein as the outcome. For the reverse MR analysis, instrumental variables for AML were selected using a genome-wide significance threshold of P<5×10 ^-8^. If no sufficient instruments were available at this threshold, a more lenient threshold of P<5×10^–6^ was applied to obtain an adequate number of SNPs for analysis. To account for multiple testing, the false discovery rate (FDR) correction was applied; a causal association was considered significant if the adjusted p-value (PFDR) < 0.05, and suggestive evidence was noted for nominal significance (P < 0.05). All analyses were performed using the R package “TwoSampleMR” (v0.5.6).

### 2.4 Pathway and functional enrichment analysis

We performed enrichment analysis on the plasma proteins identified through MR to investigate their potential biological functions and involvement in pathways. All analyses were conducted in metascape online tool (https://metascape.org) and visualizing the results.

### 2.5 Bayesian colocalization analysis

Bayesian colocalization analysis was performed using the “coloc” package with default parameters to estimate the probability that two traits share the same causal variant. This approach helps determine whether observed associations are due to causal effects of genetic variants on traits, rather than being influenced by LD or other confounding factors. The colocalization analysis evaluates five hypotheses: H0: Neither the exposure nor the outcome is associated with the genomic region; H1: The exposure is associated with the genomic region, but the outcome is not; H2: The outcome is associated with the genomic region, but the exposure is not; H3: The exposure and the outcome are associated with the genomic region through different SNPs; H4: The exposure and the outcome are associated with the genomic region through a shared SNP. We calculated the posterior probabilities (PP) for each hypothesis and determined the presence of colocalization evidence for a protein based on the condition PPH3 + PPH4 > 0.5 (21).

### 2.6 Protein structure and specific expression analysis

Firstly, we utilized UniPort (https://www.uniprot.org/) to investigate the structure and cellular localization of proteins, and analyzed the tissue-specific expression of proteins through GeneCards (https://www.genecards.org/). Then, we analyzed the scRNA data through the Single Cell Portal database (https://singlecell.broadinstitute.org/) to evaluate the gene expression differences of TCL1A, TNFSF8, WFDC1 and TNFAIP8 in different cell types. The access number of the dataset is SCP1987, which includes bone marrow mononuclear cells from 42 patients with acute myeloid leukemia and 10 healthy individuals without specific immune cell enrichment or removal of blast cells. This dataset contains 254,910 cells and 33,947 genes (22). Finally, we analyzed the gene expression patterns of each subtype of AML based on the ELN and WHO guidelines using the BeatAML database (http://www.vizome.org/aml2/). Group comparisons were performed using one-way ANOVA or Kruskal-Wallis tests based on data distribution normality, with appropriate *post-hoc* tests (Tukey’s or Dunn’s test) for multiple comparisons.

### 2.7 ELISA validation

To validate the robustness of the MR results, we collected serum samples from 6 age and sex-matched healthy controls and 20 AML patients (12 newly diagnosed patients, 4 in complete remission (CR) after treatment, and 4 with relapse following treatment, the detail information was list in **Supplementary Table 2**). The TNFSF8 ELISA kit (catalog number EH0120) was purchased from FineTest (https://www.fn-test.com/, China), the TNFAIP8 kit (catalog number ELH-TNFAIP8) was obtained from RayBiotech (https://www.raybiotech.com/, USA); and the WFDC1 and TCL1A kits (catalog numbers E9351h and E1709h, respectively) were sourced from EiAab (https://www.eiaab.com.cn/, China). The levels of TCL1A, TNFSF8, WFDC1, and TNFAIP8 in the serum were measured according to the manufacturer’s instructions for each respective kit. Briefly, all serum sample measurements were performed in triplicate. The mean value was used for subsequent analysis unless the coefficient of variation (CV) exceeded 15%, in which case the measurement was repeated. The intra-assay and inter-assay CVs for each target protein were below 10% and 15%, respectively. Standard curves were generated for each assay, all of which demonstrated excellent linearity (R² > 0.99). The limits of detection and quantification were determined for each kit according to the manufacturer’s protocols. All laboratory personnel were blinded to the clinical status of the samples during the measurement process. Any outliers were identified using the ROUT method (Q = 1%) and were excluded from the final analysis. Group comparisons were performed using one-way ANOVA or Kruskal-Wallis tests based on data distribution normality, with appropriate *post-hoc* tests (e.g., Tukey’s or Dunn’s test) for multiple comparisons.

### 2.8 Correlation analysis between potential protein and clinical parameters

Clinical data from 12 newly diagnosed AML patients were collected to analyze the correlation between plasma protein levels of TNFAIP8, TCL1A, WFDC1, and TNFSF8 and the clinical characteristics of the patients. Pearson correlation analysis was performed, and a p-value < 0.05 was considered statistically significant.

### 2.9 Phenome-wide association study

PheWAS was performed to evaluate the pleiotropic effects of potential therapeutic targets and possible adverse effects. The outcome involved obtaining phenotypic data from the Finnish database in version R10, encompassing 2408 phenotypes categorized into 46 groups. This extensive dataset was employed for phenome-wide MR analysis. P < 0.05 was considered statistically significant.

### 2.10 Potential drug target

The target genes were submitted to the Drug Signatures Database (DSigDB, http://dsigdb.tanlab.org/DSigDBv1.0/) to explore potential interactions between the proteins and available drugs. The target genes were input into the Enrichr gene set enrichment analysis platform (https://maayanlab.cloud/modEnrichr/) to access the DSigDB database and predict potential drug candidates that could interact with the genes of interest.

## 3 Results

### 3.1 MR analysis

**Figure 2:**
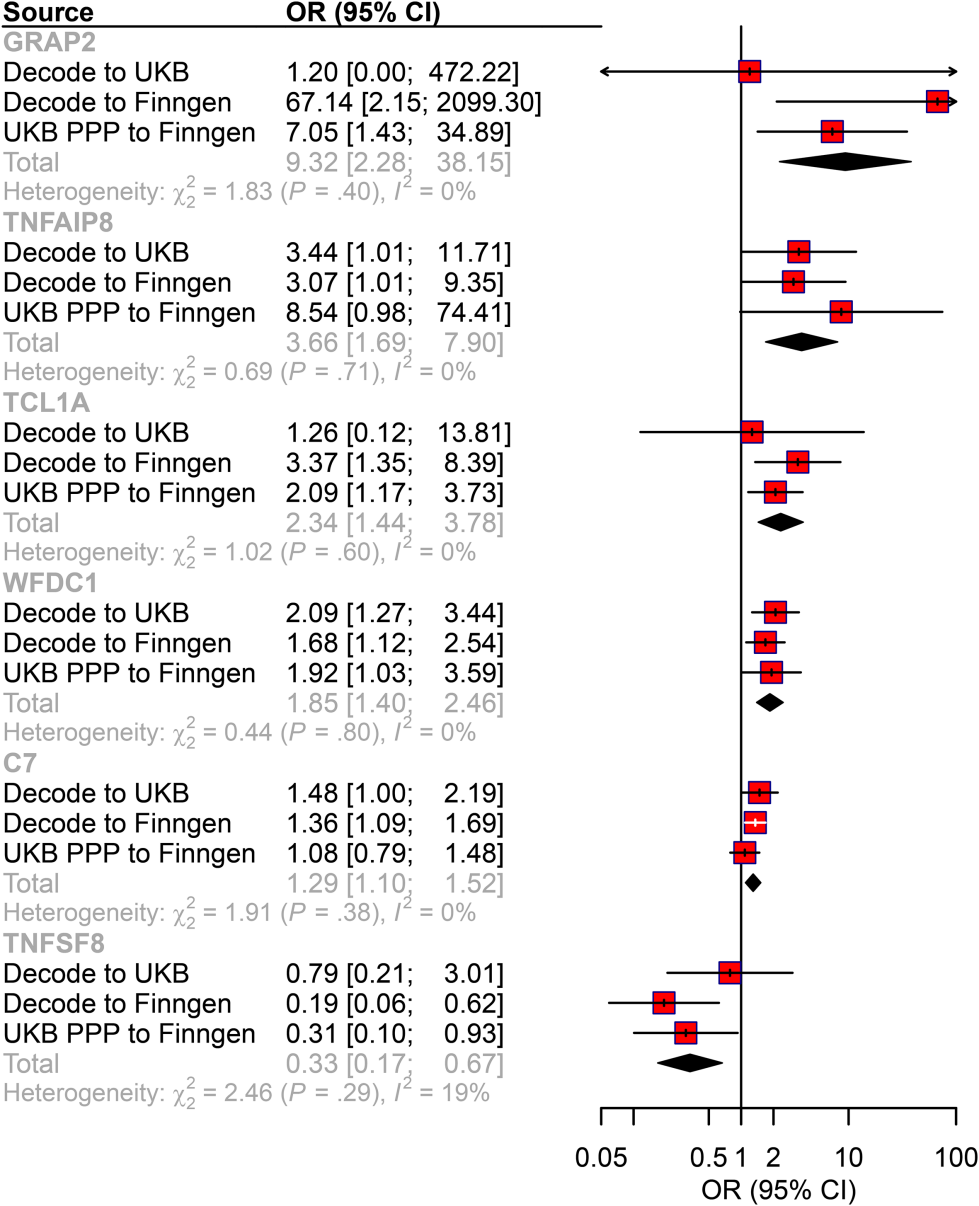
The causal relationship between plasma proteins and AML risk in the MR analysis. Forest plot of the MR analysis. CI: confidence interval; OR: odds ratio.

To systematically identify therapeutic targets, we integrated multi-center data from exposure-outcome pairs. Three independent analyses identified 65, 69, and 86 AML-associated plasma proteins (p<0.05) from deCODE to FinnGen, deCODE to UK Biobank, and UKB-PPP to FinnGen, respectively (**Supplementary Figures S1A-C**; **Supplementary Table 3**). Enrichment analysis revealed that these proteins were associated with immune and inflammatory processes (**Supplementary Figures S1D-F**). We then screened for proteins consistently detected in at least two analyses with the same directional effects across all three datasets. This approach identified six candidate proteins: GRAP2, TNFAIP8, TCL1A, WFDC1, C7, and TNFSF8 (**Figure 2**). In addition, in the reverse MR analysis for six candidate proteins, no causal association were found (**Supplementary Table 4**). Notably, five proteins exhibited risk-promoting effects for AML progression (GRAP2 OR = 9.32, 95% CI: 2.28-38.151; TNFAIP8 OR = 3.66, 95% CI: 1.69-7.90; TCL1A OR = 2.34, 95% CI: 1.44-3.78; WFDC1 OR = 1.85, 95% CI: 1.40-2.46; C7 OR = 1.29, 95% CI: 1.10-1.52);, while TNFSF8 showed protective potential (OR = 0.33, 95% CI: 0.17-0.67). Further, we compared the top cis-pQTLs (the most significant SNPs associated with protein levels) for each key protein between the deCODE (SomaScan) and UKB-PPP (Olink) datasets. We observed high consistency in the top cis-pQTLs for four of the six proteins: WFDC1 (rs400345 in both platforms), TCL1A (rs78986913 in both platforms), TNFAIP8 (rs1035376 in both platforms) GRAP2 (rs148328786 in both platforms). For TNFSF8 and C7, the top cis-pQTL differed between platforms (TNFSF8, rs1006026 in deCODE vs. rs10081728 in UKB-PPP) (C7, rs72758315 in UKB-PPP, rs79534924 in deCODE).

### 3.2 Bayesian colocalization analysis

**Figure 3:**
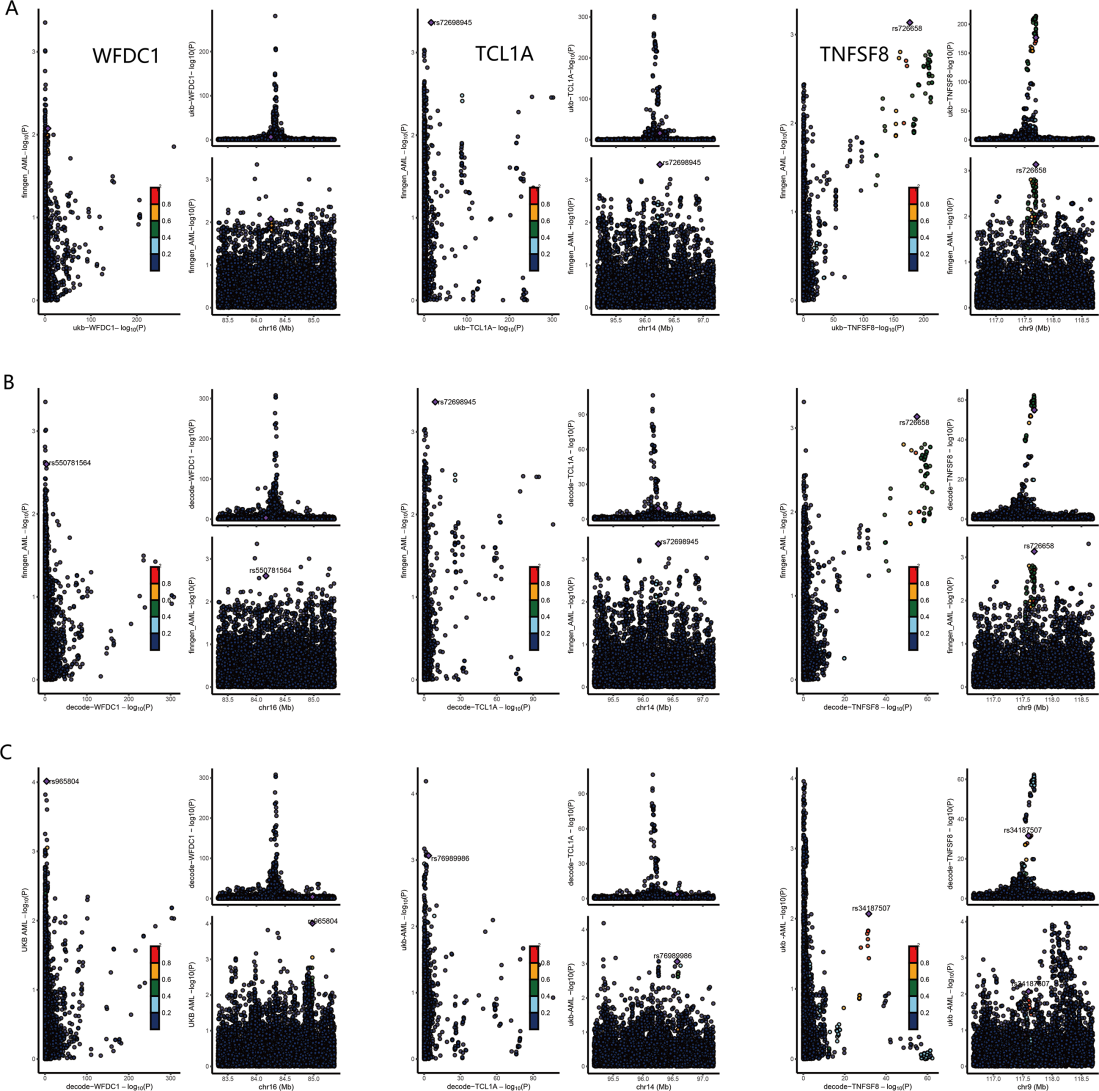
Colocalization analysis results for WFDC1, TCL1A and TNFSF8. **(A)** results for UKB-PPP to FinnGen. **(B)** results for deCODE to FinnGen. **(C)** results for deCODE to UK Biobank.

To validate the genetic causality of these candidate proteins, we performed Bayesian colocalization analysis. The results suggested that TCL1A (PPH3+PPH4 = 59.65, 51.22, 64.99), TNFSF8 (PPH3+PPH4 = 68.62, 61.06, 71.63), WFDC1 (PPH3+PPH4 = 52.10, 65.62, 62.41), and TNFAIP8 (PPH3+PPH4 = 55.46, 53.92, 55.35) shared causal variants with AML across all three analyses (deCODE to FinnGen, deCODE to UK Biobank, and UKB-PPP to FinnGen, **Figure 3**). However, the PPH3+PPH4 values for GRAP2 (49.49, 38.80, 38.39) and C7 (53.06, 46.86, 51.32) indicated weak colocalization with AML in the genome. The detail results of colocalization analysis were list in **Supplementary Table 5**.

### 3.3 Biological relevance of these proteins

**Figure 4:**
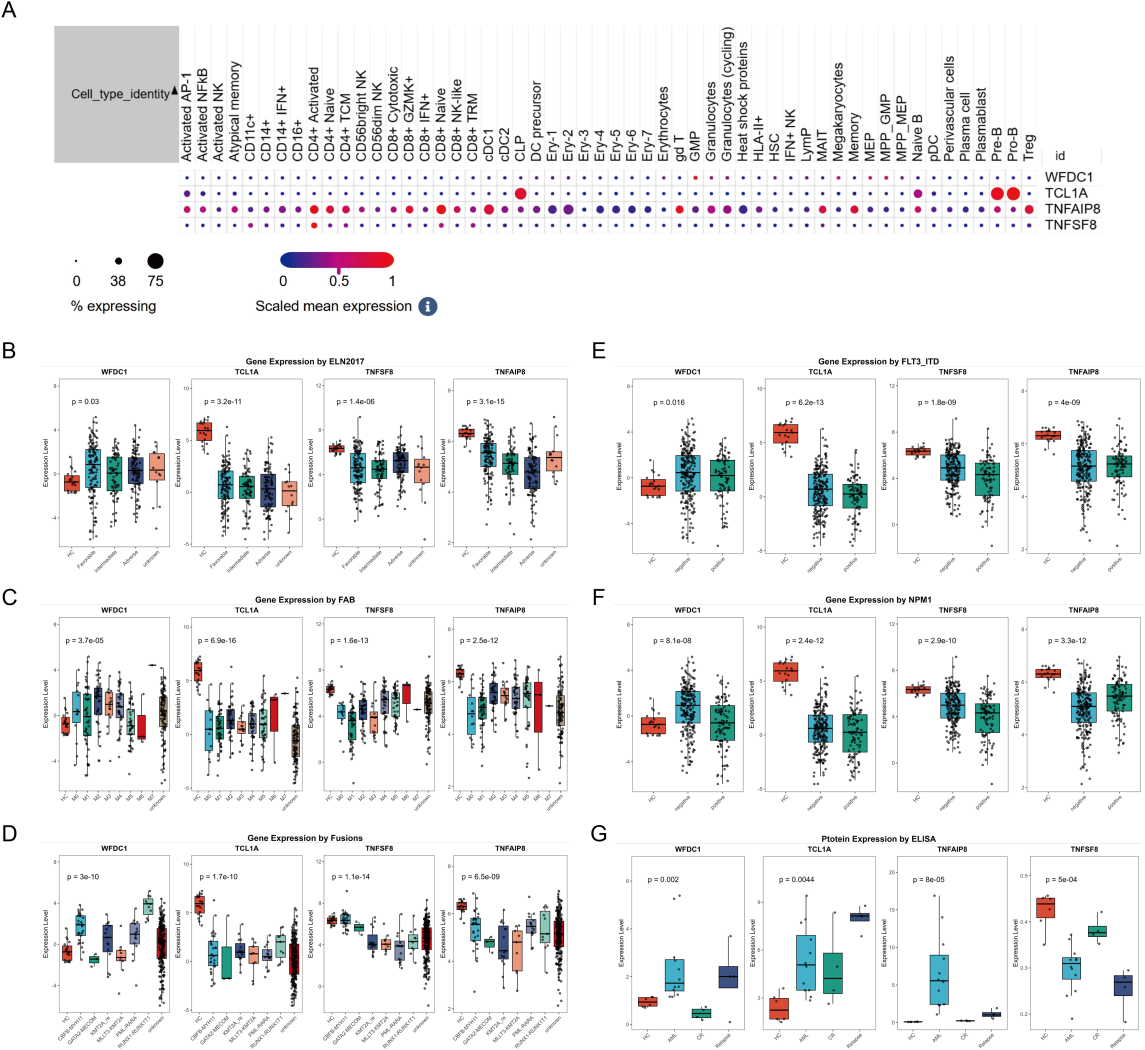
The expression of WFDC1, TCL1A, TNFAIP8 and TNFSF8 in single cell level **(A)**; The expression of WFDC1, TCL1A, TNFAIP8 and TNFSF8 based on ELN 2017 **(B)**, FAB subtype **(C)**, fusion gene **(D)**, FLT3-ITD status **(E)** and NPM1 status **(F)**. Serum expression levels of WFDC1, TCL1A, TNFAIP8 and TNFSF8 in different groups, including healthy controls (HC), newly diagnosed acute myeloid leukemia (AML) patients, AML patients in complete remission (CR) after treatment, and relapsed AML patients after treatment **(G)**.

Having established genetic associations, we next investigated the biological relevance of these proteins. The structure of protein was shown in **Supplementary Figure S2A**, and subcellular localization of the protein indicated that TNFSF8 is a cell-surface receptor (membrane), WFDC1 is a secreted protein, TCL1A is an intracellular protein (cytoplasm; endoplasmic reticulum; microsome; nucleus), and TNFAIP8 is also an intracellular protein (cytoplasm) (**Supplementary Figure S2B**). Tissue expression profiles showed that WFDC1, TCL1A and TNFAIP8 were mainly localized in immune cells and blood components, unfortunately, there is no data on TNFSF8 protein in GeneCards (**Supplementary Figure S3**). Moreover, scRNA-seq results revealed that WFDC1 was mainly detected in GMP, MEP and MPP; TCL1A was mainly expressed in Pre-B cells and Pro-B cells; TNFAIP8 was primarily expressed in CD4+ T cells, CD8+ T cells, while TNFSF8 was predominantly expressed in CD4+ T cells (**Figure 4A**). Finally, we analyzed the expression of these genes in the AML subtypes defined by the ELN and WHO guidelines. The expression of TNFAIP8 was related to ELN2017 (**Figure 4B**). Their expressions varied among different FAB subtypes (**Figure 4C**). WFDC1 might be specific to RUNX1-RUNXT1, and TNFSF8 might be specific to CBFB-MYH11 (**Figure 4D**). TCL1A had lower expression in FLT3-ITD-positive patients, WFDC1 and TNFSF8 had lower expression in NPM1-positive patients, while TNAFIP8 had higher expression in both FLT3-ITD and NPM1-positive patients (**Figures 4E, F**).

### 3.4 ELISA analysis and correlation analysis of plasma proteins

We further verified the above results using ELISA, and ELISA results confirmed significantly elevated expression levels of TNFAIP8, WFDC1 and TCL1A, and low TNFSF8 expression in AML patients compared to healthy controls (P<0.05). Notably, in AML patients who achieved CR after treatment, the expression levels of TNFAIP8 and WFDC1 decreased, the expression levels of TNFSF8 increased, while TCL1A showed no significant change. Furthermore, TNFAIP8 levels were significantly higher and TNFSF8 levels were significantly lower in relapsed patients compared to CR patients, while the other proteins showed no significant differences (**Figure 4G**). Finally, to assess clinical significance of these proteins, we collected clinical data from 12 newly diagnosed AML patients and analyzed the correlation between plasma TNFAIP8, TCL1A, WFDC1, and TNFSF8 protein levels and patient clinical features. However, no significant correlations were found between the expression levels of these plasma proteins and the clinical parameters (**Supplementary Figure S4**).

### 3.5 Phenome-wide association study

The above results indicated these four proteins could use as therapeutic target; to assess the potential beneficial or harmful effects of the four AML-associated plasma proteins on other phenotypes, we performed a phenome-wide association study (PheWAS). The results were shown in **Supplementary Figure S5** and **Supplementary Table 6**. These associations highlight both therapeutic potential and possible off-target effects of modulating these proteins.

### 3.6 Candidate drug prediction

**Figure 5:**
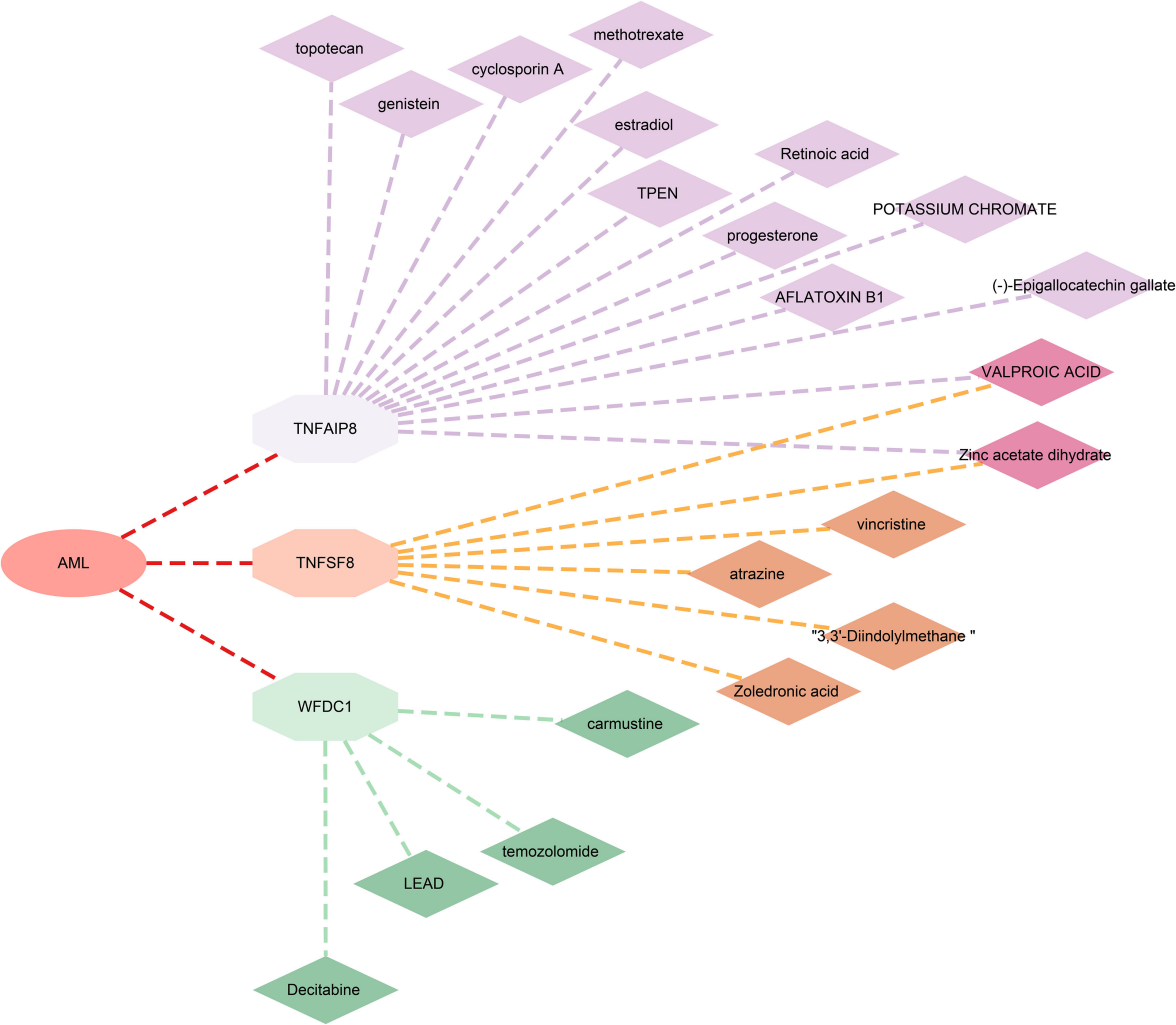
Drugs targeting identified significant proteins.

Furthermore, to evaluate the potential of the identified proteins as drug targets, we investigated their interactions with known drugs using the DSigDB database. We found 13 drugs associated with TNFAIP8, 6 with TNFSF8, and 4 with WFDC1. However, no related drugs were identified for TCL1A (**Figure 5**; **Supplementary Table 6**).

## 4 Discussion

This study represents the first proteome-wide Mendelian randomization (MR) analysis to systematically investigate causal relationships between plasma proteins and AML risk. We identified four plasma proteins with robust genetic evidence supporting their roles in AML pathogenesis: TNFAIP8, TCL1A, and WFDC1 as risk factors, and TNFSF8 as a protective factor. Notably, TNFAIP8 and TNFSF8 exhibited dynamic expression patterns correlated with disease relapse, suggesting their potential as biomarkers for monitoring therapeutic response.

To ensure the robustness of our findings, we integrated multi-center datasets from deCODE, UKB-PPP, FinnGen, and UK Biobank, conducting three independent MR analyses (deCODE-to-FinnGen, deCODE-to-UKB, UKB-PPP-to-FinnGen). We prioritized proteins with consistent directional effects across all analyses. Platform concordance analysis further confirmed high consistency in top cis-pQTLs for four proteins between SomaScan and Olink, Although TNFSF8 and C7 showed differing top cis-pQTLs between platforms, their causal effect directions remained consistent. Additionally, Steiger filtering validated the causal direction, and reverse MR analyses ruled out potential reverse causation for all four proteins, reinforcing the genetic basis of these associations. Bayesian colocalization analysis was conducted using a threshold of PPH3 + PPH4 > 0.5 to identify regions with evidence of shared genetic association. While this approach effectively detects regional colocalization, we acknowledge that it may introduce potential bias by prioritizing loci where protein and disease associations are driven by distinct but linked causal variants (high PPH3) rather than a single shared variant (high PPH4) (23). Among the candidates, four proteins including TNFAIP8, TCL1A, WFDC1, and TNFSF8 emerged with compelling genetic and multi-omics support. Although this stringent, multi-evidence intersection approach enhanced the reliability of our findings, it may have excluded proteins with weaker yet biologically relevant signals.

WFDC1 performs multiple important biological functions, including protease activity, calcium ion transport, and bacterial growth (24–26). Our result indicated WFDC1 is a secreted protein primarily expressed in hematopoietic progenitors (GMP, MEP, MPP). Notably, we found that WFDC1 expression was particularly elevated in patients with the RUNX1-RUNX1T1 fusion, suggesting its potential role as a subtype-specific biomarker or therapeutic target in this AML subgroup. Although widely recognized as a tumor suppressor in various solid tumors (24–26), our results indicate that it may play an opposite, potentially tumor-promoting role in AML. This functional divergence may be attributed to differences in tumor microenvironment composition, particularly regarding cancer-associated fibroblasts (25), which exhibit distinct characteristics in hematological malignancies compared to solid tumors (27). The significant discrepancy in WFDC1’s proposed roles across cancer types warrants further investigation into the underlying tissue-specific mechanistic switches.

TCL1A is an intracellular protein (cytoplasm, endoplasmic reticulum, nucleus). scRNA-seq confirmed its predominant expression in Pre-B and Pro-B cells, aligning with its known B-cell association (28, 29). However, our findings reveal its potential role as a risk factor in AML. Notably, emerging evidence indicates that TCL1A overexpression serves as a negative prognostic indicator not only in B-cell malignancies (27) but also in various solid tumors (30–32), where it promotes tumor progression through modulating immune microenvironment and enhancing cell survival pathways. This consistent oncogenic role across malignancies suggests that TCL1A may similarly function as a disease-promoting factor in AML, possibly through mechanisms involving immune modulation within the bone marrow microenvironment.

TNFAIP8 demonstrated the most significant association with increased AML risk. This finding aligns with previous studies linking TNFAIP8 to chemotherapy resistance in AML via activation of the ERK pathway (33). TNFAIP8 mRNA expression was correlated with ELN risk stratification, and our experimental validation further confirmed elevated TNFAIP8 levels in AML patients, particularly in relapsed cases, indicating its potential utility as both a diagnostic biomarker and therapeutic target. The dynamic changes in TNFAIP8 expression during disease progression and treatment response underscore its involvement in AML pathophysiology and its possible role in mediating treatment resistance. .

While previous studies reported elevated TNFSF8 expression in M4 and M5 AML subtypes (34, 35), which was consistent with our mRNA-level findings. Our ELISA results showed reduced protein levels in AML patients, this discrepancy may be attributed to following reasons: First, the relatively small proportion of M4/M5 subtypes in our validation cohort may have underrepresented these specific subtypes. Second, it is important to note that our MR analysis utilized GWAS data that did not distinguish between AML subtypes, which may have influenced the overall causal estimates and contributed to the observed discrepancies. More importantly, our MR results demonstrating a protective effect of TNFSF8 against AML development provide genetic evidence for its potentially protective role, which may operate through mechanisms distinct from its expression patterns. Nevertheless, the protective role of TNFSF8 in AML should be taken with caution. This also highlights the complex relationship between protein expression levels and causal effects, where MR identifies genetically determined causal relationships that may not always correlate with observed expression patterns due to post-translational modifications, regulatory feedback mechanisms, or tissue-specific processing.

From a drug development perspective, TNFSF8 is a well-characterized cell-surface receptor (membrane), representing a classic, directly ligandable target amenable to antibody-based therapies. WFDC1 is a secreted protein, making it directly targetable by neutralizing antibodies or recombinant decoy receptors. TCL1A is an intracellular protein (cytoplasm; endoplasmic reticulum; microsome; nucleus) that functions as a non-enzymatic scaffold and co-activator, representing an indirectly ligandable target where modulation would require challenging intracellular targeting strategies (e.g., small molecules, PROTACs). TNFAIP8 is also an intracellular, non-enzymatic scaffold protein (cytoplasm), similarly representing an indirectly ligandable target with high mechanistic complexity for drug development. Drug analyses identified 13 compounds targeting TNFAIP8, WFDC1, or TNFSF8, including FDA-approved agents such as decitabine and vincristine. We supposed that decitabine may synergize with WFDC1 inhibition to remodel microenvironment (36, 37); while vincristine could modulate TNFSF8-related immune dysregulation (38, 39). Further investigation into the interactions between these targets and existing drugs will not only help elucidate novel mechanisms of drug action but also inform new combination strategies for precision therapy in AML.

Importantly, PheWAS data indicated favorable safety profiles for these targets, mitigating concerns about off-target effects. However, the preliminary nature of clinical correlations derived from our limited cohort (n=20) necessitates cautious interpretation. Larger prospective studies should evaluate the prognostic significance of these proteins across molecular subtypes and treatment modalities.

Although our integrated multi-omics approach has provided novel insights into causal plasma proteins in AML, several limitations of this study must be acknowledged. First, the European ancestry of the analyzed cohorts may limit the generalizability of our findings to other populations, highlighting the need for validation in ethnically diverse cohorts. Second, the scarcity of associations meeting stringent FDR thresholds (PFDR < 0.05) likely reflects the limited statistical power of current AML GWAS, though our integrative analytical framework partially mitigated this by requiring cross-cohort consistency. Third, we were unable to perform AML subtype stratification due to insufficient subtype annotations in the large-scale datasets used. Given the well-established molecular heterogeneity of AML under ELN and WHO classifications, this limitation may obscure potential subtype-specific protein signatures. Future studies incorporating well-annotated, subtype-specific cohorts are essential to elucidate the clinical utility of these biomarkers within distinct AML subgroups. Fourth, in our colocalization analysis, we used a combined probability threshold (PPH3 + PPH4 > 0.5) to identify regions with evidence of shared genetic association. While this approach is effective for detecting regional colocalization, it may also retain loci where protein and disease associations are driven by distinct but linked causal variants (high PPH3), potentially introducing false positives. Future studies focusing on fine-mapping and functional validation will be necessary to distinguish between these scenarios.

Despite these limitations, the dynamic changes observed in TNFAIP8 and TNFSF8 during treatment and relapse suggest their potential value for monitoring disease status. We propose that future studies leverage longitudinal protein measurements and machine learning approaches to develop predictive models for relapse risk and therapeutic response. Such efforts could significantly advance personalized management and treatment strategies for AML.

## 5 Conclusion

By integrating MR, single-cell omics, and experimental validation, this study establishes TNFAIP8, TCL1A, WFDC1, and TNFSF8 as novel causal mediators of AML. These proteins offer dual utility as biomarkers and therapeutic targets, with TNFAIP8 and TNFSF8 particularly promising for relapse prediction. Collaborative efforts to develop targeted inhibitors or agonists, coupled with mechanistic investigations, will be critical to advance these findings toward clinical application.

## Data Availability

The raw data supporting the conclusions of this article will be made available by the authors, without undue reservation.

## Supplementary material

Supplementary Figure 1 Volcano plots of the MR analysis(A–C, deCODE to FinnGen, deCODE to UK Biobank, and UKB-PPP to FinnGen, respectively). GO enrichment analysis of plasma proteins identified in the MR analysis (D–F, deCODE to FinnGen, deCODE to UK Biobank, and UKB-PPP to FinnGen, respectively).

Supplementary Figure 2 The structure (A) and subcellular localization (B) of WFDC1, TCL1A, TNFAIP8 and TNFSF8.

Supplementary Figure 3 Tissue expression profiles for WFDC1, TCL1A, and TNFAIP8.

Supplementary Figure 4 The correlations between the expression levels of these plasma proteins and clinical parameters.

Supplementary Figure 5 Manhattan plot for MR phenome-wide association study of (A)TCL1A, (B) TNFAIP8, (C) TNFSF8 and (D) WFDC1. A dot represents a disease or trait.

Supplementary Table 1 The information for selected IV.

Supplementary Table 2 Clinical information for patients and healthy control.

Supplementary Table 3 MR analysis results.

Supplementary Table 4 Reverse MR analysis results.

Supplementary Table 5 Colocalization analysis results.

Supplementary Table 6 Phenome-wide association study analysis results.

Supplementary Table 7 Protein-drug interaction analysis for potential drug targets.
