# Supplementary figures and images for: Identification and validation of plasma protein biomarkers as therapeutic targets in acute myeloid leukemia: an integrative multi-omics study
Source: Front Immunol. 2025 Oct 22;16:1659811. doi: 10.3389/fimmu.2025.1659811 (PMC12586143; doi:10.3389/fimmu.2025.1659811)

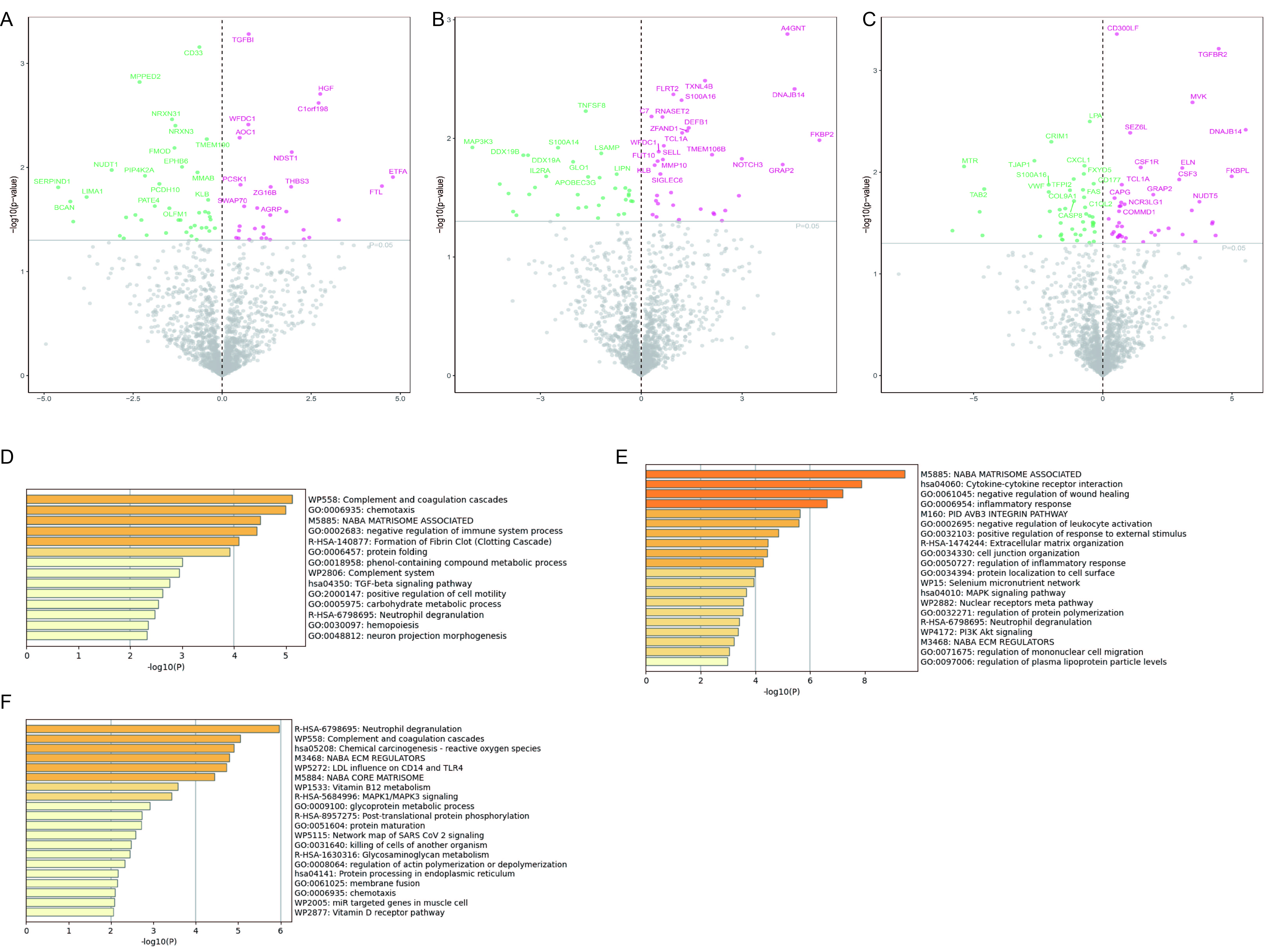

Supplement: Supplementary Figure 1 — Volcano plots of the MR analysis(A–C, deCODE to FinnGen, deCODE to UK Biobank, and UKB-PPP to FinnGen, respectively). GO enrichment analysis of plasma proteins identified in the MR analysis (D–F, deCODE to FinnGen, deCODE to UK Biobank, and UKB-PPP to FinnGen, respectively). [file Image1.jpeg]

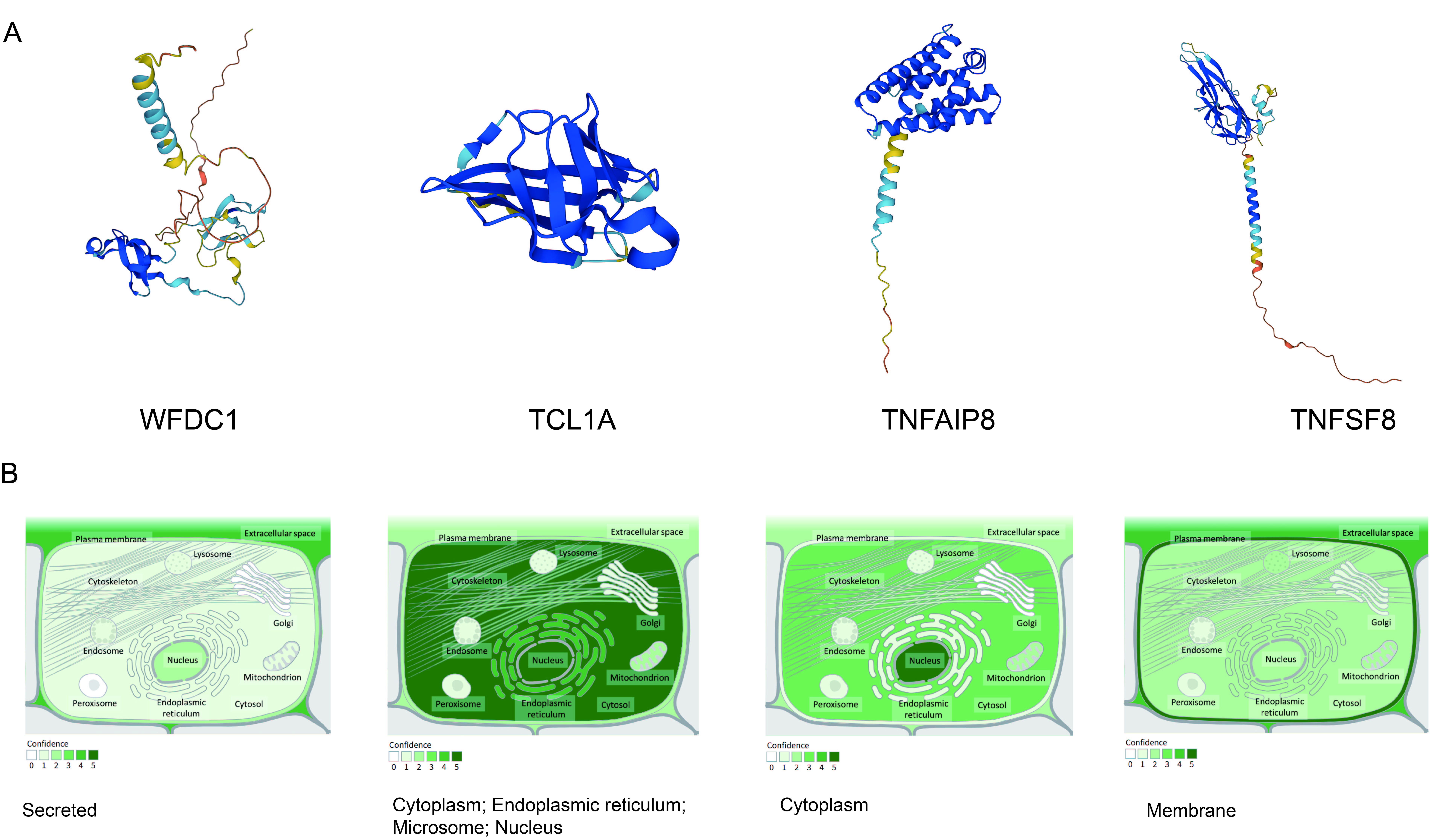

Supplement: Supplementary Figure 2 — The structure (A) and subcellular localization (B) of WFDC1, TCL1A, TNFAIP8 and TNFSF8. [file Image2.jpeg]

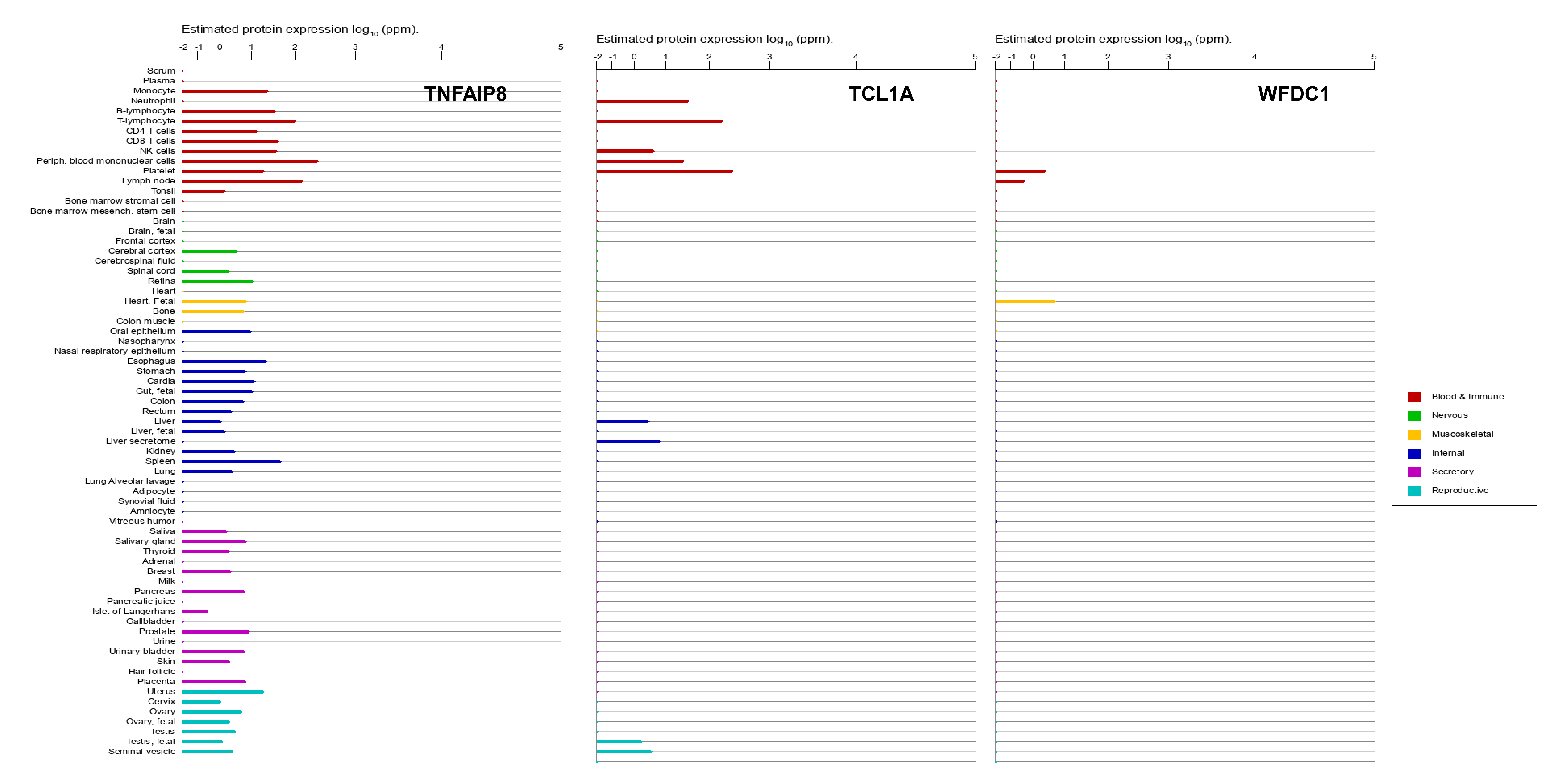

Supplement: Supplementary Figure 3 — Tissue expression profiles for WFDC1, TCL1A, and TNFAIP8. [file Image3.jpeg]

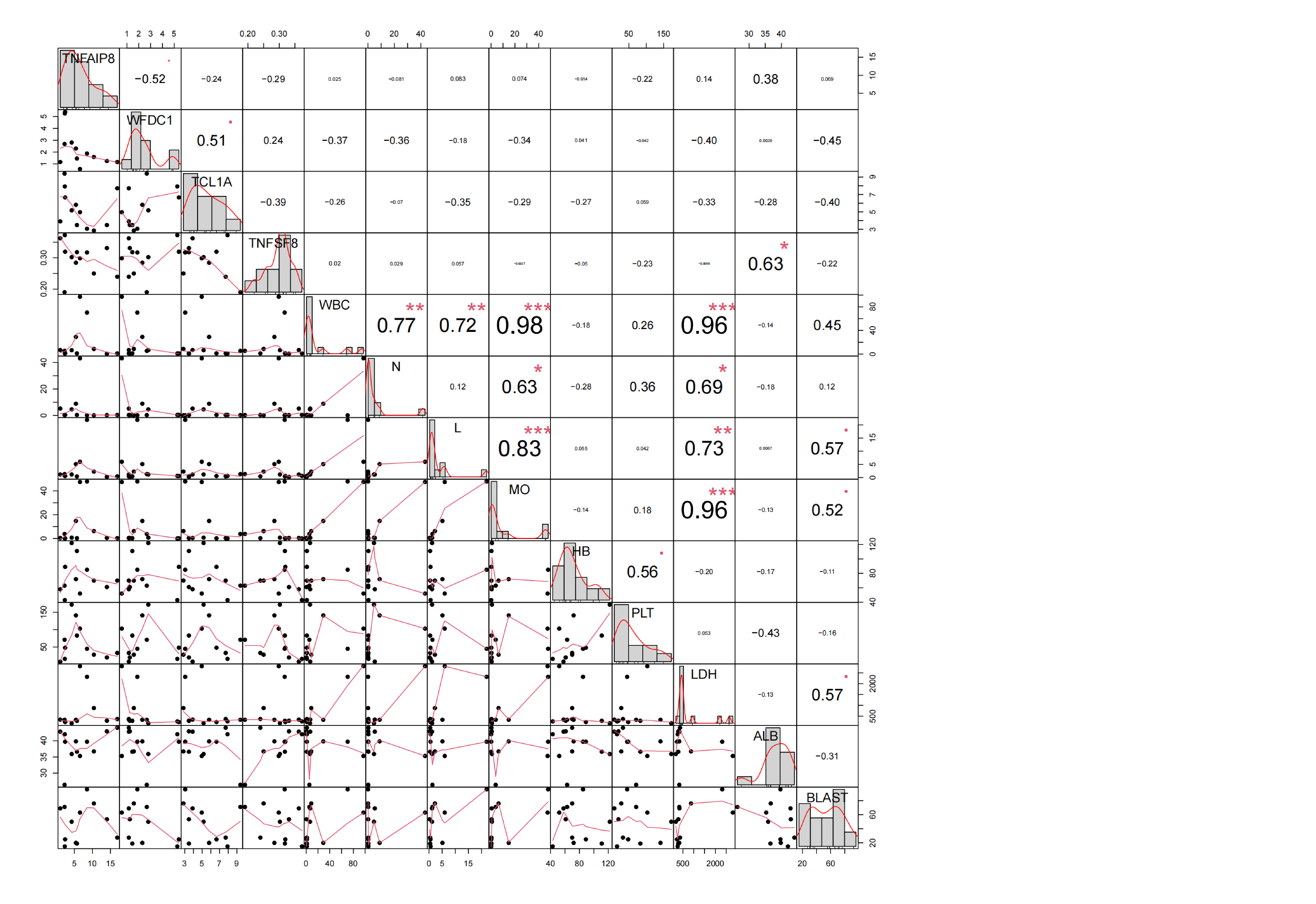

Supplement: Supplementary Figure 4 — The correlations between the expression levels of these plasma proteins and clinical parameters. [file Image4.jpeg]
